# Reaction of Human Monoclonal Antibodies to SARS-CoV-2 Proteins With Tissue Antigens: Implications for Autoimmune Diseases

**DOI:** 10.3389/fimmu.2020.617089

**Published:** 2021-01-19

**Authors:** Aristo Vojdani, Elroy Vojdani, Datis Kharrazian

**Affiliations:** ^1^ Department of Immunology, Immunosciences Laboratory, Inc., Los Angeles, CA, United States; ^2^ Department of Preventive Medicine, Loma Linda University School of Medicine, Loma Linda, CA, United States; ^3^ Regenera Medical, Los Angeles, CA, United States; ^4^ Department of Neurology, Harvard Medical School, Boston, MA, United States; ^5^ Department of Neurology, Massachusetts General Hospital, Charlestown, MA, United States

**Keywords:** COVID-19, SARS-CoV-2, cross-reactivity, molecular mimicry, autoimmunity

## Abstract

We sought to determine whether immune reactivity occurs between anti-SARS-CoV-2 protein antibodies and human tissue antigens, and whether molecular mimicry between COVID-19 viral proteins and human tissues could be the cause. We applied both human monoclonal anti-SARS-Cov-2 antibodies (spike protein, nucleoprotein) and rabbit polyclonal anti-SARS-Cov-2 antibodies (envelope protein, membrane protein) to 55 different tissue antigens. We found that SARS-CoV-2 antibodies had reactions with 28 out of 55 tissue antigens, representing a diversity of tissue groups that included barrier proteins, gastrointestinal, thyroid and neural tissues, and more. We also did selective epitope mapping using BLAST and showed similarities and homology between spike, nucleoprotein, and many other SARS-CoV-2 proteins with the human tissue antigens mitochondria M2, F-actin and TPO. This extensive immune cross-reactivity between SARS-CoV-2 antibodies and different antigen groups may play a role in the multi-system disease process of COVID-19, influence the severity of the disease, precipitate the onset of autoimmunity in susceptible subgroups, and potentially exacerbate autoimmunity in subjects that have pre-existing autoimmune diseases. Very recently, human monoclonal antibodies were approved for use on patients with COVID-19. The human monoclonal antibodies used in this study are almost identical with these approved antibodies. Thus, our results can establish the potential risk for autoimmunity and multi-system disorders with COVID-19 that may come from cross-reactivity between our own human tissues and this dreaded virus, and thus ensure that the badly-needed vaccines and treatments being developed for it are truly safe to use against this disease.

## Introduction

Coronavirus disease (COVID-19) has become one of the greatest global public health concerns of our century. The COVID-19 pandemic has placed an immediate call to action for medical researchers to investigate how SARS-CoV-2 can impact the worldwide human population. While, naturally, the search for a successful vaccine and efficient treatment protocols are paramount, immunologists who focus on autoimmunity have been concerned whether the infection or even a newly developed vaccine itself can trigger autoimmunity *via* cross-reactivity. Cross-reactivity occurs when amino acid sequence homology exists between a pathogen and self-tissue proteins (1). In this mechanism, antibodies formed against SARS-CoV-2 would also bind to human tissue proteins leading to autoimmune reactivity. An insufficiently vetted vaccine might mean trading freedom from COVID-19 to an autoimmune assault in the future.

There are three important questions regarding the role of cross-reactivity with SARS-CoV-2. First, does cross-reactivity play a role in the multi-system disorders associated with SARS-CoV-2 infection? Second, how does cross-reactivity contribute to the pathophysiology of SARS-CoV-2–induced autoimmunity? Third, are there any concerns for autoimmune development with either infection or vaccination with SARS-CoV-2?

We will begin with the first question of whether cross-reactivity can be involved in the multi-system response of COVID-19 infection. We believe the answer is probable, since some of the systemic disease clinical manifestations of COVID-19 cannot be explained solely by the binding of SARS-CoV-2 spike proteins with cell membranes of tissues that exhibit angiotensin-converting enzyme 2 (ACE2). For example, a significant deadly expression of the infection is the development of disseminated intravascular coagulopathy. Coagulopathy has become a key indicator of mortality in infected subjects (2). In a recent correspondence in the New England Journal of Medicine, the serology of infected patients suffering from coagulopathy demonstrated significantly elevated levels of anti-cardiolipin and anti–β2-glycoprotein autoantibodies (3). These findings suggest the possibility of autoimmune reactivity that may be part of the SARS-CoV-2 pathophysiological sequela. It is possible that some of the clinical manifestations of central nervous system, skin, gastrointestinal, and organ diseases may also be associated with autoimmune reactions.

The second important question is whether SARS-CoV-2 infection can lead to cross-reactivity. The development of pathogen-induced cross-reactivity requires two key criteria. First, the viral pathogen must exhibit sequence homology with human tissue proteins, and second, there must be loss of immune tolerance (4). Lyons-Wieler recently mapped out the immunogenic epitopes of SARS-CoV-2 proteins and compared them to human proteins in search of patterns of significant homologous matching in order to establish the possibility of viral-induced autoimmunity. He identified substantial cross-reactive mapping with many SARS-CoV-2 spike and nuclear proteins to human tissue protein sequences (5). There have also been several findings of immune dysregulation associated with loss of immune tolerance with COVID-19 infection. Giamarellos-Bourboulis described complex immune dysregulation in COVID-19 patients with severe respiratory failure (6). The unique pattern of immune dysfunction included: immune dysregulation or major decrease in HLA-DR14 on monocytes; macrophage activation syndrome; and lower absolute count for CD3+/CD4+/CD45+ T-lymphocytes, CD3−/CD16+/CD56+/CD45+ NK cells, and CD19+/CD45+ B-lymphocytes among patients with COVID-19 when compared to healthy subjects. These immunological shifts in combination with SARS-CoV-2 amino acid sequence homology mapping with human tissue proteins orchestrate a combination of immune variables that suggest cross-reactivity can potentially occur with patients infected with SARS-CoV-2.

The third important question to consider is whether cross-reactivity between COVID-19 and human tissue can lead to autoimmune disease development either from the infection or directly from vaccination. Determining this can be an enormous task because the development of most autoimmune diseases may take 3 to 18 years (7). Segal and Shoenfeld have raised concerns for vaccine-induced autoimmunity by citing examples of how previous vaccinations have induced cross-reactive autoimmunity in susceptible subgroups. They cite specific examples of how vaccine-induced cross-reactivity has led to the onset of systemic lupus erythematous, demyelinating autoimmune diseases, narcolepsy, and postural orthostatic tachycardia syndrome (8). In a very interesting letter, Kanduc and Shoenfeld addressed the issue of peptide sharing between SARS-CoV-2 spike glycoprotein and lung-surfactant-related proteins (9). They suggested that because the SARS-CoV-2 and lung surfactant proteins shared 13 out of 24 pentapeptides, the immune response following infection with SARS-CoV-2 may lead to cross-reactions with pulmonary surfactant proteins, followed by SARS-CoV-2–associated lung disease (9). Furthermore, very recently they presented indisputable proof of molecular mimicry as a potential mechanism for contributing to SARS-CoV-2 associated diseases (10). Based on their findings, they warned against the use of the entire SARS-CoV-2 antigens in the vaccines and cautioned that perhaps the use of only unique peptides would be the most effective way to fight the SARS-CoV-2 infection. Due to the significant red flags for the potential cross-reactivity between SARS-CoV-2 and human tissue, we have undertaken to study the interaction of antibodies made against SARS-CoV-2 spike protein, nucleoprotein, envelope protein and membrane protein with various autoimmune target proteins associated with many serious diseases. This way, we can establish the potential risk for autoimmunity and multi-system disorders with COVID-19 that may come from cross-reactivity between our own human tissues and this dreaded virus, and thus ensure that the badly-needed vaccines and treatments being developed for it are truly safe to use against this pandemic.

## Materials and Methods

### Ethical Guidelines

We purchased human monoclonal and rabbit polyclonal antibodies from certified, regulated commercial sources who use immunization protocols for the animals that conform to The Guide for the Care and Use of Laboratory Animals published by the National Institutes of Health, publication no 85-23, 1985

### Antibody and Antigens

Human IgG1 monoclonal antibody made against SARS-CoV-2 spike protein S1 and S2 domains was purchased from Novus Biologicals (Centennial, CO USA). Human IgG1 monoclonal antibody made against SARS-CoV-2 nucleoprotein was obtained from The Native Antigen Company (Langford Locks, Oxfordshire, UK). Rabbit IgG polyclonal antibody against SARS-CoV-2 envelope and membrane proteins was purchased from Antibodies Online Inc. (Limerick, PA USA).

### Proteins

Recombinant SARS-CoV-2 spike protein S1 subunit, recombinant SARS-CoV-2 nucleocapsid protein, envelope proteins, and membrane proteins were purchased from RayBiotech (Atlanta, GA, USA).

Glial fibrillary acidic protein, brain-derived neurotrophic factor (BDNF), myoglobin, platelet glycoprotein, alpha-synuclein, acetylcholine receptor, lysosome, and elastase were purchased from Calbiochem (San Diego, CA, USA).

Parietal cell antigen, intrinsic factor, fibrinogen, laminin, thyroid peroxidase (TPO), thyroglobulin (TG), myeloperoxidase, collagen type V, and neuraminidase were purchased from MP Biologicals (Solon, OH, USA).

Cardiolipin, actin, myelin basic protein (MBP), tropomyosin, ganglioside GM1, insulin, liver microsomes, transglutaminases (tTGs), enolase, beta-amyloid protein, tau protein, somatotropin, human serum albumin (HSA), and dipeptidylpeptidase were purchased from Sigma-Aldrich (St Louis, MO, USA).

Different peptides of occludin, zonulin, claudin 5 and 6, beta-catenin, aquaporin-4 (AQP4), presenilin, fibulin, protein disulfide isomerase, cerebellar, enteric nerve neuronal nuclear antigen, glutamate-R, dopamine-R, insulin-R, and glutamic acid decarboxylase 65 (GAD-65), all with purity greater than 90%, were synthesized by Biosynthesis (Lewisville, TX, USA).

Mitochondria M2 antibody kit was purchased from Trinity Biotech (Jamestown, NY, USA).

From Inova Diagnostics (San Diego, CA, USA) we purchased plates coated with purified F-actin antigen; nuclei and nucleoli of HEP-2 cell plus individual antigens; SS-A, SS-B, Sm/RNP, Scl-70, centromere, PCNA, and Jo1 (all of which were used for nuclear antigen or NA); purified Sm, RNP, SS-A, SS-B, Scl-70, and Jo1) all of which were used for extractable nuclear antigen or ENA); and calf thymus DNA, which was used for dsDNA.

The 55 antigens that were selected provide a wide net to evaluate key autoimmune target proteins that include skin, gastrointestinal, pancreas, liver, heart, muscle, joint, thyroid, brain, enteric nerve, tight junction proteins and cellular components. Our selection was inspired by the human tissues used in the earlier Lyons-Weiler study (5), which was the first to report significant homology between those human tissues and SARS-CoV-2 proteins. Although there is some overlap, we selected our tissue antigens, first, because they are involved with the extra-pulmonary manifestations of COVID-19, and, second, because they reflect key target proteins involved with common autoimmune diseases.

Human sera were obtained from Innovative Research prior to 2020. These sera were screened for the presence of mitochondria M2 antibody using the Trinity Biotech Mitochondria M2 antibody kit. From the many screened sera we selected four that tested negative for M2 antibody and four that tested positive for M2 antibody. These four negative sera and four positive sera, in conjunction with the calibrators, negative controls and positive controls from the mitochondria M2 antibody kit, were applied to the SARS-CoV-2 spike proteins and nucleoproteins.

### Reaction of Anti–SARS-CoV-2 Spike Protein, Nucleoprotein, Envelope Protein, and Member Protein Antibodies With Different Tissue Antigens

In addition to ready-to-use microwell plates coated with different tissue antigens, peptides and proteins, including recombinant SARS-CoV-2 spike proteins, nucleoproteins, envelope and membrane proteins, at a concentration of 1 mg/mL were diluted 1:100 in 0.01 M phosphate buffer saline (PBS) at pH 7.4. 100 microliters containing 1 microgram of each antigen was added to a series of 96-well microtiter plates.

After incubation for 8 h at room temperature (RT) and 18 h at 4°C, plates were washed three times using an ELISA washer, and 200 microliters of 2% BSA + 2% dry milk were added to each well and incubated for 24 h at 4°C in order to block the non-specific binding of the antibody to the antigen-coated wells. To examine the binding of SARS-CoV-2 antibodies to each one of these antigens, 100 microliters of human anti-spike protein and human anti-nucleoprotein at optimal dilutions of 1:200 – 1:400, and rabbit anti-envelope and rabbit anti-membrane proteins at a dilution of 1:200 were each added to quadruplicate wells of microtiter plates coated with antigens. After 1 h incubation and washing, optimal dilution of alkaline phosphatase-labeled anti-human or anti-rabbit IgG was added to the appropriate sets of plates, which were then incubated again for 1 h at room temperature (RT). For the removal of unbound antibodies, plates were washed five times and 100 microliters of substrate para-nitro-phenyl-phosohate were added, and color development was measured after 30 min using an ELISA reader at 405 nm. The means of the respective quadruplicate wells were calculated and used in the graphs.

The percentage of tissue reaction with each antibody was calculated based on the following formula:

$$\%\;\text{of reaction with the antibody}=\frac{\text{OD of tissue reactivity}-\mathrm{OD of background}}{\text{OD of SARS-CoV-}2\;\text{reactivity}\;-\;\text{OD of background}}$$

To determine the specificity of human monoclonal and rabbit polyclonal antibodies binding to different tissue antigens, these antibodies were replaced with the same dilution of human serum from a healthy subject or with non-immunized rabbit serum and added to quadruplicate wells. Furthermore, the antibodies and other reagents were added to four wells coated with 2% HSA and four wells coated with 2% BSA alone; these were then used as negative controls. After the addition of other reagents to these control wells, the ODs were measured.

### Binding of Serially Diluted Antibodies Against SARS-CoV-2 Spike Protein, Nucleoprotein, Envelope Protein, and Membrane Protein to Tissue Antigens

For the demonstration of the specificity of SARS-CoV-2 antibodies binding to different tissues, 4 sets of 5 different strips of ELISA plate, each containing 8 wells, were coated respectively with SARS-CoV-2 antigens or different tissue antigens. These antigens were chosen because they showed moderate to strong immune reactivity with either SARS-CoV-2 spike protein, nucleoprotein envelope protein or membrane protein antibodies. The SARS-CoV-2 antibodies were serially diluted ranging from 1:200 to 1:25,600 and were then added to each antigen-coated well. After incubation, washing, the addition of secondary antibodies, and the completion of other required ELISA steps, the ODs were recorded at 405 nm.

### Inhibition of SARS-CoV-2 Spike Protein, Nucleoprotein, Envelope Protein, and Membrane Protein Antibodies Binding to Various Cross-Reactive Tissue Antigens in the Presence of the Same Antigens

The inhibition study in an ELISA assay is for the purpose of proving that the reaction of the antibody to an antigen is specific. This is done by the addition of increasing concentrations of a specific antigen to an antibody in different test tubes first, and the subsequent addition of this mixture to ELISA plate wells coated with the same antigen.

For example, to prove that the binding of anti-SARS-CoV-2 spike protein human monoclonal antibody to M2 protein is specific, the following steps were taken:

1. Eight different wells of an ELISA plate were coated with a different pre-determined optimal concentration of M2 protein.
2. 100 microliters of human monoclonal antibody to SARS-CoV-2 spike protein was then added to each of 8 different tubes.
3. We did not add any M2 antigen to the first tube (#1) containing SARS-CoV-2 spike protein monoclonal antibody; this tube served as the baseline control showing the degree of uninhibited binding of SARS-CoV-2 spike protein monoclonal antibody to M2 protein. The other 7 tubes received increasing concentrations of M2 protein. Tube #2 received 2 micrograms, tube #3 received 4 micrograms, and tubes # 4, 5, 6, 7 and 8 received 8, 16, 32, 64 and 128 micrograms of M2 antigen respectively.
4. The contents of the 8 tubes, now containing SARS-CoV-2 spike protein monoclonal antibody and from 0 to 128 micrograms of M2 antigen, were individually mixed, and the mixed contents of each tube were then added to the 8 different wells coated with M2 protein described in Step #1.

After incubation, washing, and the addition of anti-human IgG labeled with enzyme and the completion of all ELISA steps, the ODs were recorded at 405 nm, and the inhibition of anti-SARS-CoV-2 antibody binding to M2 protein was demonstrated graphically in proportion to the increased concentration of M2 protein in the test tubes containing the SARS-CoV-2 antibody.

All of these steps were similarly followed for inhibition study with the SARS-CoV-2 nucleoprotein, envelope protein, and membrane protein antibodies, and with antigens such as MBP, GAD-65, actin, insulin-R, and intestinal epithelial cell.

### Amino Acid Sequence Similarity Between SARS-CoV-2 Spike Protein, Nucleoprotein and Mitochondrial M2 Protein, and F-actin

We used the NIH/US National Library of Medicine’s BLAST (Basic Local Alignment Search Tool) sequence matching program to study the degrees of possible mimicry or amino acid (AA) sequence similarities between SARS-CoV-2 spike, nucleoprotein, and other proteins with M2 protein (11), F-actin (12) and thyroid peroxidase (TPO) (12).

### Reaction of Sera Containing No Levels, Low Levels, or High Levels of Mitochondrial Antibodies With Mitochondrial M2 Antigen and SARS-CoV-2 Spike Proteins and Nucleoproteins

Using a commercially available kit from Trinity Biotech for the measurement of antibodies in patients with primary biliary cirrhosis and associated disorders, we first measured the presence of M2 antibody in four control sera and in serum from four individuals who had tested positive for M2 antibody. We then applied the kit’s negative control calibrator, low and high positive controls, plus the four negative sera and the four sera positive for M2 antibody to an ELISA plate coated with both SARS-CoV-2 spike proteins and nucleoproteins. Following the kit’s instructions, the ELISA steps were completed, and ODs were obtained.

### Statistical Analysis

Statistical analysis was performed by comparing the ODs obtained for the reactive tissue antigens with the mean OD of non-reactive tissue antigens + 3SD using STATA 14.2 software. Independent t-tests were performed to evaluate mean differences of optical densities between controls and antigens. A Bonferroni adjustment was conducted to account for type 1 errors with multiple comparisons and alpha was set to < 0.001.

## Results

In this study, we measured the degree of immune reactivity of human monoclonal antibody made against SARS-CoV-2 spike protein and nucleoprotein and rabbit polyclonal antibody made against SARS-CoV-2 envelope and membrane proteins with 55 different tissue proteins and peptides. Using ELISA methodology, we first found that human serum from a healthy subject and unimmunized rabbit serum did not react with spike protein, nucleoprotein, envelope protein, membrane protein, or with the 55 different tissue proteins and peptides. The ELISA ODs for all these reactions were within 3 SD above the mean of the control values, or OD < 0.25 (**Table 1**).

**Table 1:** % Reactivity of SARS-CoV-2 Spike, Nucleoprotein, Envelope and Membrane Protein Antibodies with the Same Proteins and Different Cross-Reactive Tissue Antigens.

Antigens	Spike protein OD	% reactivity	Nucleoprotein OD	% reactivity	Envelope protein OD	% reactivity	Membrane protein OD	% reactivity
SARS-CoV-2	3.40	100 ++++	3.76	100 ++++	3.68	100 ++++	3.78	100 ++++
Actin	0.74	17.6 +	1.1	27.1 ++	0.78	18.0 +	0.95	22.2 +
Mitochondrial antigen (M2)	1.52	41.8 +++	1.94	50.1 +++	2.03	53.4 +++	2.58	67.0 ++++
ENA	0.85	21.0 +	0.44	9.0 +	0.53	11.0 +	0.21	2.2 –
NA	1.34	36.2 ++	0.97	23.6 +	0.13	0.0 –	0.46	8.8 +
Histone	0.65	14.8 +	0.92	22.2 +	0.27	3.7 –	0.78	17.6 +
S100B	0.46	9.0 +	0.87	20.8 +	0.23	2.5 –	0.44	8.2 +
MBP	0.53	11.1 +	0.55	12.0 +	0.41	7.6 +	0.32	4.9 –
NFP	1.98	56 ++++	0.42	8.5 +	0.27	3.7 –	2.0	51.1 +++
Synapsin	0.81	19.8 +	0.95	23.0 +	0.17	0.08 –	0.28	3.8 –
Beta-amyloid P	0.83	20.4 +	0.82	19.4 +	0.18	1.1 –	0.36	6.0 +
Tau protein	0.41	7.4 +	0.23	3.3 –	0.18	1.1 –	0.28	3.8 –
Collagen	0.45	8.6 +	0.65	14.8 +	0.18	1.1 –	0.85	19.2 +
Alpha-myosin	0.72	17.0 +	0.89	21.3 +	0.36	6.2 +	0.94	21.2 +
Tropomyosin	0.21	1.2 –	0.25	3.8 –	0.25	3.1 –	0.58	12.1 +
TPO	0.98	25.1 ++	0.95	23.0 +	0.26	3.3 –	0.96	22.5 +
Liver microsome	1.00	25.7 ++	1.00	24.4 +	0.17	0.08 –	0.30	4.4 –
PDH peptide	0.91	22.9 +	0.94	22.7 +	0.22	2.2 –	0.64	13.7 +
GAD-65	1.35	36.5 ++	1.08	26.5 ++	0.15	0.03 –	0.43	7.9 +
Insulin	0.25	2.5 –	0.16	0.4 –	0.15	0.03 –	0.97	22.8 +
Insulin-R	0.72	17.0 +	1.77	45.5 +++	0.21	1.9 –	0.52	10.4 +
Phospholipid	0.93	23.5 +	0.83	19.7 +	0.37	6.5 +	0.95	22.2 +
tTG-6	0.58	12.7 +	0.44	9.0 +	0.17	0.08 –	0.20	1.6 –
tTG-3	0.42	23.2 +	0.92	22.2 +	0.18	1.1 –	0.23	2.4 –
tTG-2	0.79	19.2 +	0.72	16.7 +	0.24	2.8 –	0.25	3.0 –
Int epi cells	0.49	9.9 +	0.46	9.6 +	2.13	56.0 ++++	0.95	22.2 +
Beta-catenin	0.95	24.1 +	0.56	12.3 +	0.24	2.8 –	0.15	0.3 –
Claudin	0.33	4.9 –	0.26	4.1 –	0.15	0.03 –	0.17	0.8 –
Occ + zon	0.72	17.0 +	1.32	33.0 ++	0.61	13.2 +	1.12	26.9 ++
27 other tissues*	0.34	5.2 –	0.36	6.8 –	0.30	4.5 –	0.32	4.9 –

Percentage of reactivity of tissue antigen(s) with a SARS-CoV-2 antibody was calculated based on the formula given in the Results section. Percentages above the established cutoff point for a particular SARS-CoV-2 antibody were considered significant, and the reaction percentages were further classified according to strength as follows: 0 to percentage based on antibody-specific cutoff point = – or insignificant; above cutoff-based percentage to 25% = + or weak; 25.1–40% = ++ or moderate; 40.1–55% = +++ or strong; >55% = ++++ or very strong.

*The 27 other tissues are glial fibrillary acidic protein, brain-derived neurotrophic factor, myoglobulin, platelet glycoprotein, alpha-synuclein, acetylcholine receptor, lysosome, elastase, parietal cell antigen, intrinsic factor, laminin, thyroglobulin, myeloperoxidase, neuraminidase, ganglioside GM_1_, enolase, somatotropin, dipeptidylpeptidase, aquaporin-4, presenilin, fibulin, protein disulfide isomerase, cerebellar, enteric nerve neuronal nuclear antigen, glutmate-R, dopamine-R, dsDNA.

As was expected, the strongest reactions were between anti-SARS-Cov-2 spike protein antibody and SARS-CoV-2 spike protein (OD 3.40 or very strong), anti-SARS-Cov-2 nucleoprotein antibody and SARS-CoV-2 nucleoprotein (OD 3.76 or very strong), anti-SARS-Cov-2 envelope protein antibody and SARS-CoV-2 envelope protein (OD 3.68 or very strong), and anti-SARS-Cov-2 membrane protein antibody and SARS-CoV-2 membrane protein (OD 3.78 or very strong), which is close to the maximum detection limit of the assay (OD 4.0). These SARS-CoV-2 antibodies reacted from low to very high with 28 out of 55 tissue antigens. These 28 antigens were a diverse collection of tissue groups that included gut and barrier proteins, gastrointestinal system cells, thyroid, nervous system, heart, joint, skin, muscle, mitochondria and liver tissues, and antigens used for the screening of autoimmune diseases. The mean OD of the anti-SARS-Cov-2 spike protein antibody’s reactivity with the non-reactive 27 tissue antigens +3SD was 0.34.

**Figure 1:**
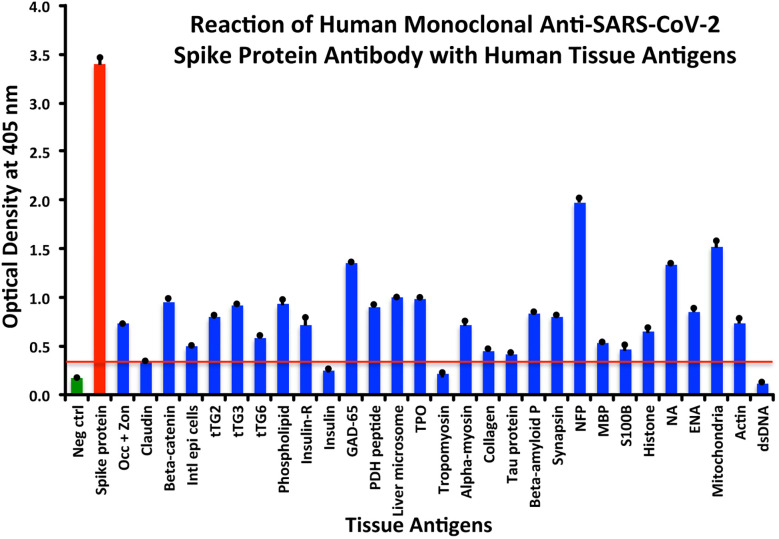
Reaction of anti-SARS-CoV-2 spike protein human monoclonal antibody with human tissue antigens. Each bar represents the calculated mean out of four different values for the same antigen. The mean OD of the anti-SARS-Cov-2 spike protein antibody’s reactivity with the non-reactive 27 tissue antigens +3SD was 0.34, which was used as the cutoff point, represented by the red line. Everything above this cutoff point is significant.

Using this 0.34 OD as a cutoff, we found that human anti-SARS-Cov-2 spike protein antibody reacted strongest with neurofilament protein or NFP (OD 1.98), followed by strong reactions with M2 (OD 1.52), GAD-65 (OD 1.35), and nuclear antigen or NA (OD 1.34). The reaction of this spike protein antibody with TPO and liver microsome was moderate (ODs 0.98, 1.0). With another 19 antigens the spike antibody’s reaction was weak with ODs ranging from 0.41 to 0.85 (see **Figure 1**).

**Figure 2:**
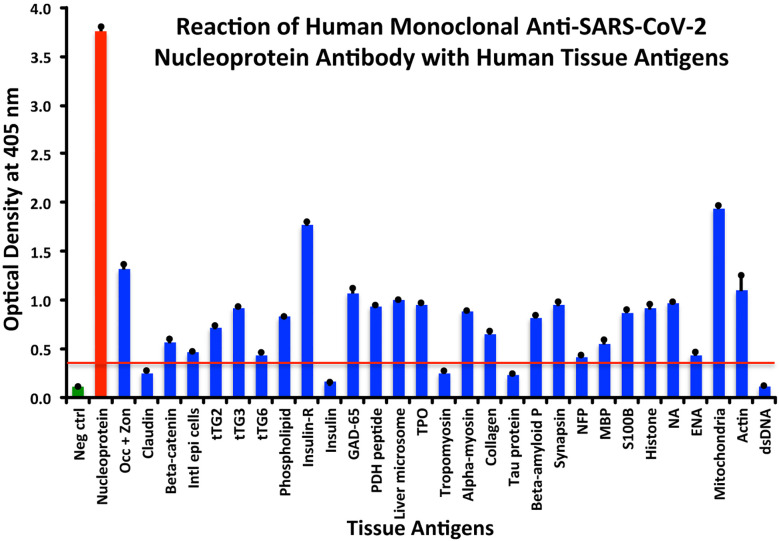
Reaction of anti-SARS-CoV-2 nucleoprotein human monoclonal antibody with human tissue antigens. Each bar represents the calculated mean out of 4 different values for the same antigen. The mean OD of the anti-SARS-Cov-2 nucleoprotein antibody’s reactivity with the non-reactive 31 tissue antigens +3SD was 0.36, which was used as the cutoff point, represented by the red line. Everything above this cutoff point is significant.

Using the proper cutoff point for this antibody (0.36 OD), human monoclonal antibody to nucleoprotein showed from weak to very strong reactivity with 24 out of the 55 tested antigens. These were 24 of the same 25 antigens with which the spike protein antibody reacted, and although there were some variations, in general, the reactions of the nucleoprotein antibody were comparable to those of the spike protein. The nucleoprotein antibody had the strongest reactions with M2 (OD 1.94) and insulin-R (OD 1.77), and a strong reaction with occludin+zonulin (OD 1.32). Reactions with GAD-65 and actin were moderate (ODs 1.08, 1.1) while the reactions with the 20 other tissue antigens were weak, ranging from 0.42 to 0.97 (see **Figure 2**).

**Figure 3:**
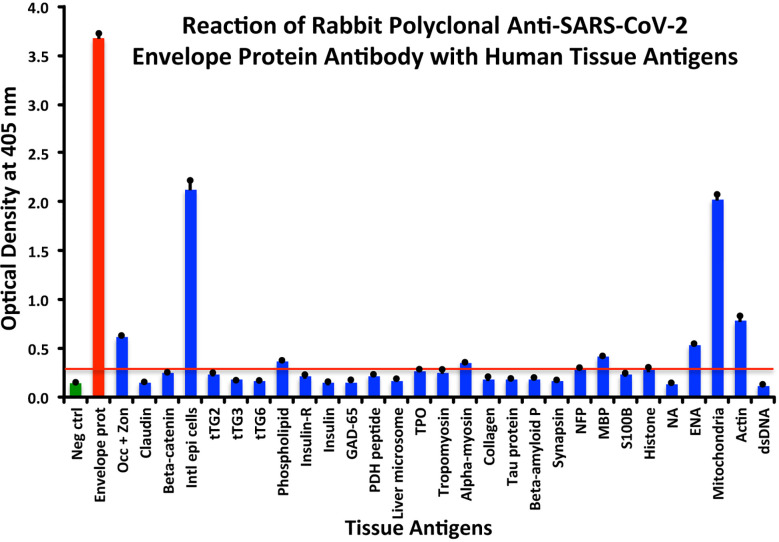
Reaction of anti-SARS-CoV-2 envelope protein rabbit polyclonal antibody with human tissue antigens. Each bar represents the calculated mean out of 4 different values for the same antigen. The mean OD of the anti-SARS-Cov-2 envelope protein antibody’s reactivity with the non-reactive 47 tissue antigens +3SD was 0.30, which was used as the cutoff point, represented by the red line. Everything above this cutoff point is significant.

In comparison with the other antibodies, at a cutoff of 0.30 OD, SARS-CoV-2 envelope protein antibody’s interaction with the 55 different tested antigens showed significant results for only 8 antigens, resulting in very strong reactions only with M2 (OD 2.03) and intestinal epithelial cell antigens (OD 2.13). The reactivity with actin (OD 0.78), occludin+zonulin (OD 0.61), ENA (OD 0.53) MBP (OD 0.41), phospholipid (OD 0.37) and alpha-myosin (OD 0.36) is considered low. The reactions for envelope protein antibody with the other 47 different antigens were statistically insignificant or negative (see **Figure 3**).

**Figure 4:**
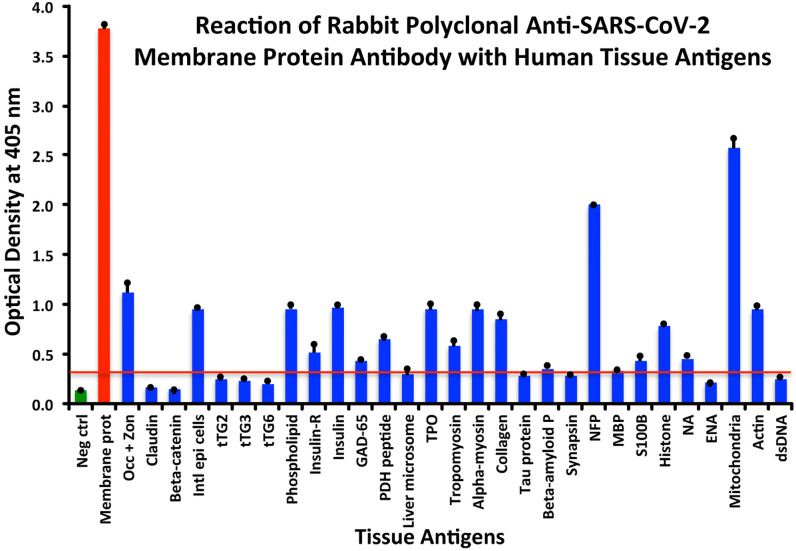
Reaction of anti-SARS-CoV-2 membrane protein rabbit polyclonal antibody with human tissue antigens. Each bar represents the calculated mean out of 4 different values for the same antigen. The mean OD of the anti-SARS-Cov-2 membrane protein antibody’s reactivity with the non-reactive 37 tissue antigens +3SD was 0.32, which was used as the cutoff point, represented by the red line. Everything above this cutoff point is significant.

At a cutoff of 0.32 OD, SARS-CoV-2 membrane protein antibody reacted with 18 out of the 55 tested antigens. The reaction was very strong with M2 (OD 2.58) and with NFP (OD 2.00). The only moderate reaction was with occludin+zonulin (OD 1.12). The reaction of this antibody with an additional 15 antigens was low (ODs of 0.36 to 0.95) (see **Figure 4**).

The reactions with dsDNA and the rest of the tissue antigens were not significantly different from the mean OD of all non-reactive tissue antigens + 3SD.

All these reactions are summarized in **Figures 1**–**4**.

The percentages of reactivity for SARS-CoV-2–specific antibody made against spike protein, nucleoprotein, envelope protein, and membrane protein with the same protein and different tissue antigens are summarized in **Table 1**.

As shown in **Table 1**, the spike protein antibody exhibited significant reactions with 25 out of 55 tested target proteins, with the strongest reactions coming from NFP and M2 antigens. The nucleoprotein antibody reacted with 24 out of 55 tissue antigens, with M2 and insulin-R showing the highest reactivity. The envelope protein antibody had weak to very strong reactions with only 8 different antigens, with the most pronounced from M2 and intestinal epithelial cell antigens. Finally, the membrane protein antibody reacted very strongly with M2 and NFP, but not as strongly with an additional 16 tissue antigens. Interestingly, M2 reacted strongly with both human monoclonal antibodies made against spike protein and nucleoprotein and with both rabbit polyclonal antibodies made against envelope and membrane proteins (**Table 1**).

### Demonstration of Analytical Specificity of Anti–SARS-CoV-2 Antibodies Binding to Human Tissue Antigens

**Figure 5:**
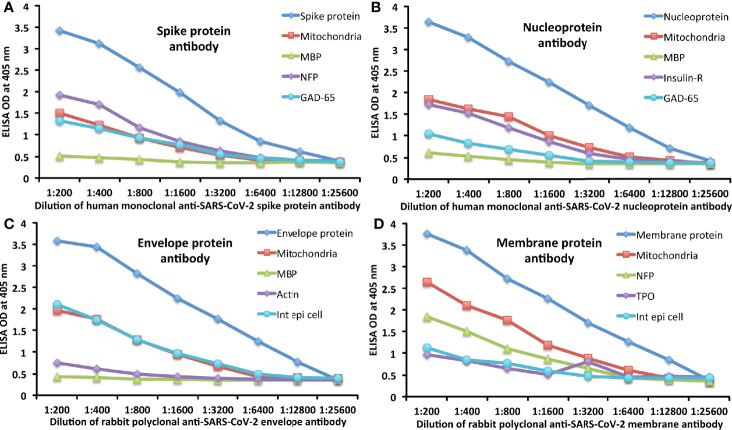
Demonstration of analytical specificity by dilution study. **(A)** Shown are the reactions of various dilutions of human monoclonal anti-SARS-CoV-2 spike protein antibody with spike protein (blue diamond ♦), M2 (red square ■), MBP (green triangle ▲), NFP (purple diamond ♦), and GAD-65 (light blue circle ●). **(B)** Shown are the reactions of various dilutions of human monoclonal anti-SARS-CoV-2 nucleoprotein antibody with nucleoprotein (blue diamond ♦), M2 (red square ■), MBP (green triangle ▲), insulin-R (purple diamond ♦), and GAD-65 (light blue circle ●). **(C)** Shown are the reactions of various dilutions of rabbit polyclonal anti-SARS-CoV-2 envelope protein antibody with envelope protein (blue diamond ♦), M2 (red square ■), MBP (green triangle ▲), actin (purple diamond ♦), and intestinal epithelial cell (light blue circle ●). **(D)** Shown are the reactions of various dilutions of rabbit polyclonal anti-SARS-CoV-2 membrane protein antibody with membrane protein (blue diamond ♦), M2 (red square ■), NFP (green triangle ▲), TPO (purple diamond ♦), and intestinal epithelial cell (light blue circle ●).

The analytical specificity of human monoclonal anti–SARS-CoV-2 spike protein antibody, human monoclonal anti-SARS-CoV-2 nucleoprotein antibody, rabbit polyclonal anti-SARS-CoV-2 envelope antibody, and rabbit polyclonal anti-SARS-CoV-2 membrane antibody was confirmed by serial dilution and inhibition studies. As shown in **Figures 5A–D**, the binding of these antibodies to 4 different SARS-CoV-2 proteins and cross-reactive antigens declined significantly in proportion to the dilution of the antibody. For example, anti-spike protein antibody reacting with spike protein at a dilution of 1:200 gave an OD of 3.4, a dilution of 1:800 gave an OD of 2.6, and a dilution of 1:25600 resulted in an OD of 0.39, which is very close to the background. The reaction of the same spike protein antibody with cross-reactive antigens such as M2, MBP, NFP, and GAD-65 also declined in proportion to the dilutions (**Figure 5A**). Similar results were obtained when serially diluted anti-nucleoprotein antibody, anti-envelope antibody, and anti-membrane antibody were applied to fixed concentrations of these same proteins or four different cross-reactive tissue antigens (**Figures 5B–D**).

**Figure 6:**
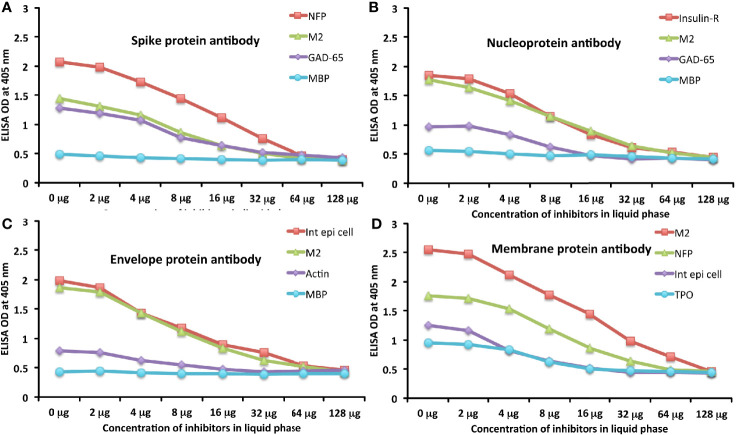
Demonstration of analytical specificity by inhibition study. **(A)** Graph shows the inhibition of human monoclonal anti-SARS-CoV-2 spike protein antibody reaction with plates coated with NFP (red square ■), M2 (green triangle ▲), GAD-65 (purple diamond ♦), and MBP (light blue circle ●) with different concentrations of the same antigen in liquid phase. **(B)** Graph shows the inhibition of human monoclonal anti-SARS-CoV-2 nucleoprotein antibody reaction with plates coated with insulin-R (red square ■), M2 (green triangle ▲), GAD-65 (purple diamond ♦), and MBP (light blue circle ●) with different concentrations of the same antigen in liquid phase. **(C)** Graph shows the inhibition of rabbit polyclonal anti-SARS-CoV-2 envelope protein antibody reaction with plates coated with intestinal epithelial cell (red square ■), M2 (green triangle ▲), actin (purple diamond ♦), and MBP (light blue circle ●) with different concentrations of the same antigen in liquid phase. **(D)** Graph shows the inhibition of rabbit polyclonal anti-SARS-CoV-2 membrane protein antibody reaction with plates coated with M2 (red square ■), NFP (green triangle ▲), intestinal epithelial cell (purple diamond ♦), and TPO (light blue circle ●) with different concentrations of the same antigen in liquid phase.

To further demonstrate the specificity of these antibody reactions, an inhibition study was performed by the addition of M2, MBP, NFP, and GAD-65 in concentrations ranging from 0 to 128 micrograms into the liquid phase of the ELISA plates that were coated with the same antigen. Compared to the baseline uninhibited reaction of the anti-spike protein antibody with the cross-reactive antigen, the addition of increased concentrations of the cross-reactive antigens, followed by the addition of primary antibody, resulted in significant inhibition of anti-spike protein antibody binding to cross-reactive tissue antigens in proportion to the degree of reactivity. For instance, this inhibition of the binding of SARS-CoV-2 spike protein antibody to NFP by NFP or M2 by M2 antigens was more pronounced than by MBP (see **Figure 6A**). A similar decline in anti-nucleoprotein antibody, anti-envelope protein antibody, and anti-membrane protein antibody binding to wells coated with the same antigens was observed when the cross-reactive tissue antigens were added to the liquid phase of the ELISA assay (**Figures 6B–D**). The decline in the ODs in proportion to the different concentrations of M2 antigen used in this inhibition study in comparison to the baseline or control tube (Tube #1) is evidence that there was specificity in the binding of the SARS-CoV-2 antibodies to the cross-reactive antigens.

### Amino Acid Sequence Similarity Between SARS-CoV-2 Proteins, Mitochondrial M2 Protein, F-actin, and TPO

Using BLAST, we did an extensive search for the degree of identity between SARS-CoV-2 proteins and mitochondrial M2 protein (human monoclonal antibody made against spike protein reacted very strongly with M2 protein), F-actin (a major component of smooth muscle, since spike protein human monoclonal antibody had moderate reactions with this antigen), and TPO (a target antigen in thyroid autoimmunity to which 2 of the monoclonal antibodies and 1 of the polyclonal antibodies had moderate reactions) (**Figure 1**). The human monoclonal antibodies for SARS-CoV-2 that we used to test for similarity with mitochondrial M2, F-actin and TPO proteins were very similar to the one used in some monoclonal antibody-based drugs that were approved for human use very recently in patients with low to moderate symptoms (13). As shown in **Tables 2**–**6**, SARS-CoV-2 proteins shared a significant number of peptide sequences with mitochondrial M2 protein, ranging from 50% to 78% identity, 58% to 63% with F-actin, and 50% to 70% with TPO. We found that some peptide sequences matched with more than one section of SARS-CoV-2; for instance, the actin sequence SIL—ASLSTF cross-reacted with the sequence SVLYNSASFSTF in the SARS-CoV-2 spike protein Chains A, B, C and E, as well as in Chain E of the SARS-CoV-2 spike receptor binding domain. An almost similar number of peptide sequences with identity percentages ranging from 33% to 49% were also detected but are not shown in the tables.

**Table 2:** Potential cross-reactive epitopes between SARS-CoV-2 spike proteins, nucleoproteins and mitochondrial M2 antigen.

SARS-CoV-2 antigen	SARS-CoV-2 sequence	Mapped start to end	Mitochondria M2 sequence	ID (%)
Chain A, Spike protein	PQCVNLTT–RT	2–11	PHCS–TTYLRT	50
Chain A, Spike protein	KEIDRLNE	14–21	KEGDKINE*	63
Chain A, Spike protein	VAETGT	27–32	VAEGGT	83
Chain A, Spike protein	QLLVPRGS	49–56	QLL—GS	63
Chain A, Spike protein	DIPIGAGIC	654–662	DVPGAIIC*	78
Chain A, Spike protein	LLQY-GS	738–743	LLQLLGS*	71
Chain A, Spike protein	IA-VEQDK	770–776	IAEVETDK*	75
Chain A, Spike protein	IKDFGGFNFSQI	794–805	IK—–NFSAI*	50
Chain A, Spike protein	IA-VEQDK	801–807	IAEVETDK	75
Chain A, Spike protein	SKRS—FI	813–818	SKISVNDFI*	56
Chain A, Spike protein	RLITGRL-QSLQT	995–1,006	RVIAQRLMQSKQT*	62
Chain A, Spike protein	LMSFPQSAPH	1,049–1,058	LMQSKQTIPH*	50
Chain A, Spike protein	AGLIA-IV	1,222–1,228	AGLITPIV	75
Chain A, Nucleoprotein	SP-RWYFY	60–66	SPGRRY-Y*	63

*This subject sequence made a match with more than one section of the SARS-CoV-2 sequence.

SARS-CoV-2 spike protein shared a significant number of peptide sequences with mitochondrial M2 protein, ranging from 50% to 78% identity. An almost similar number of peptide sequences with identity percentages ranging from 33% to 49% were also detected but are not shown in this table.

Peptide mapping was done using the NIH/US National Library of Medicine’s BLAST (Basic Local Alignment Search Tool) sequence matching program.

**Table 3:** Potential cross-reactive epitopes between SARS-CoV-2 proteins and mitochondrial M2 antigen.

SARS-CoV-2 antigen	SARS-CoV-2 sequence	Mapped start to end	Mitochondria M2 sequence
Chain i, Non-structural protein 1	LVPGFNEK	4–11	LVPADNEK
Chain i, Non-structural protein 1	VLL-RKNGNK	121–129	VLLVRKELNK
Chain i, Non-structural protein 1	DLGDELGTD	144–152	DLLAEIETD
Chain i, Non-structural protein 1	GD—ELGTD	146–152	GDLIAEVETD
Chain A, Non-structural protein 3	GIFGAD-PI	130–137	GVF-TDIPI
Chain A, Non-structural protein 7	SLLSVLLS	54–61	SMMSVTLS*
Chain A, Non-structural protein 8	LC–VD-EA	46–51	LCIIVEKEA
Chain A, Non-structural protein 9	MS–CAAGTT	15–22	MSPHCS–TT
Chain A, Non-structural protein 9	GR-FV–LA	41–46	GRVFVDPLA
Chain A, Non-structural protein 10	TMGNSTV	1–7	TMG–TV
Chain B, Non-structural protein 10	GTGQA–IT	51–57	GTGPDGRIT
Chain A, Non-structural protein 12	VSAARLTP	14–21	VSVAVSTP
Chain A, Non-structural protein 12	AAISDY-DYYR	448–457	ADISAFADY-R
Chain A, Non-structural protein 12	EDQDALF-AYT	522–531	EDIEA-FKNYT
Chain E, Non-structural protein 13	ETTADIVVFDEISMAT	369–384	ETIANDVV—SLAT*
Chain A, Helicase NSP13	ETLKAT	138–143	ETDKAT
Chain A, Helicase NSP13	SAI-NRPQ	488–494	SAIINPPQ
Chain A, Main protease	TANPKTP	95–101	TASPPTP
Chain A, Non-structural protein 16	LLVDSDLN	94–101	LLVRKELN*
Chain A, Non-structural protein 16	VSDADST—LI	104–112	VSVAVSTPAGLI*
Chain A, Protein 9b	RKTLNS-LE	58–65	RKELNKILE
Chain A, Uridylate-specific endoribonuclease	EVPV-SIINNTV	43–53	DVPIGAIICITV
Chain A, Uridylate-specific endoribonuclease	GVDIAANTVI	100–109	GVETIANDVV
Chain A, Uridylate-specific endoribonuclease	ASLNGVTL	184–191	ASMMSVTL

*This subject sequence made a match with more than one section of the SARS-CoV-2 sequence.

Other SARS-CoV-2 proteins also shared an impressive degree of identity with M2 protein. We found that some peptide sequences matched with more than one section of SARS-CoV-2.* An almost similar number of peptide sequences with identity percentages ranging from 33% to 49% were also detected but are not shown in this table.

Peptide mapping was done using the NIH/US National Library of Medicine’s BLAST (Basic Local Alignment Search Tool) sequence matching program.

**Table 4:** Potential cross-reactive epitopes between SARS-CoV-2 spike proteins, nucleoproteins and actin antigen.

SARS-CoV-2 antigen	SARS-CoV-2 sequence	Mapped start to end	Actin sequence	ID (%)
Chain A, Spike protein	GKIQDSLSST	16–25	GSILASLS-T*	60
Chain A, Spike protein	STEKSNII	85–92	STMKIKII*	63
Chain A, Spike protein	IGAGICAS	697–704	IGGSILAS*	63
Chain A, Spike protein	PS–GRLVPR	1,210–1,217	PSIVGR–P	60
Chain A, Nucleoprotein	SSSTKKS	15–21	SSSLEKS	71
Chain A, Nucleoprotein	TEGALNTPK	90–98	TEAPLN-PK	67
Chain A, Spike protein	SVLYNSASFSTF	33–44	SIL—ASLSTF**	58
Chain B, Spike protein	SVLYNSASFSTF	48–59	SIL—ASLSTF**	58
Chain C, Spike protein	SVLYNSASFSTF	48–59	SIL—ASLSTF**	58
Chain E, Spike protein	SVLYNSASFSTF	37–48	SIL—ASLSTF**	58
Chain E, Spike receptor binding domain	SVLYNSASFSTF	48–59	SIL—ASLSTF**	58

*This subject sequence made a match with more than one section of the SARS-CoV-2 sequence.

**This is an example of one sequence-to-sequence match occurring in different chains and places in the SARS-CoV-2 spike proteins.

SARS-CoV-2 spike protein shared a significant number of peptide sequences (58–63%) with F-actin. We found that some peptide sequences matched with more than one section of SARS-CoV-2; for instance, the actin sequence SIL—ASLSTF cross-reacted with the sequence SVLYNSASFSTF in the SARS-CoV-2 spike protein Chains A, B, C and E, as well as in Chain E of the SARS-CoV-2 spike receptor binding domain. An almost similar number of peptide sequences with identity percentages ranging from 33% to 49% were also detected but are not shown in this table.

Peptide mapping was done using the NIH/US National Library of Medicine’s BLAST (Basic Local Alignment Search Tool) sequence matching program.

**Table 5:** Potential cross-reactive epitopes between SARS-CoV-2 proteins and actin antigen.

SARS-CoV-2 antigen	SARS-CoV-2 sequence	Mapped start to end	Actin sequence	
Chain A, Non-structural protein 3	AE—VRTIK	3–9	AEREIVRDIK*	
Chain A, Non-structural protein 3	SSFLEMKS	165–172	SSSLE-KS	
Chain B, Non-structural protein 3	PSFLG	78–82	PSFLG*	
Chain B, Non-structural protein 8	MAIASEFSSLP-SY	1–13	MATAASSSSLEKSY	
Chain B, nsp8	SSLP-SY	7–12	SSLEKSY	
Chain B, nsp10	ANSTVLS	9–15	AN-TVLS*	
Chain A, nsp12	LMPILT	241–246	LMKILT	
Chain A, nsp12	YEAMYT-PHTVL	921–931	YEG-YALPHAIL*	
Chain A, Helicase NSP13	YIGDPAQ	400–406	YVGDEAQ*	
Chain A, Helicase NSP13	DTVSALVYDN	452–461	DDIAALVVDN*	
Chain A, Helicase NSP13	CDVTDV-TQLY	57–66	CDV-DIRKDLY	
Chain A, Helicase NSP13	PE–YFNSV	421–427	PERKY—SV*	
Chain A, 3C-like proteinase	DR-Q—TA	185–189	DRMQKEITA*	
Chain A, Papain-like protease	RE-VRTIK	3–9	REIVRDIK	
Chain A, Protein 3a	TSSIVITSGDGTT	164–176	TTGIVMDSGDGVT	
Chain A, Replicase polyprotein 1ab	DR-Q—TA	187–191	DRMQKEITA*	
Chain E, SARS-CoV-2 receptor binding domain	SVLYNSASFSTF	48–59	SIL—ASLSTF
Chain A, Uridylate-specific endoribonuclease	VDIA—ANTV	99–106	VDIRKDLYANTV
Chain A, Uridylate-specific endoribonuclease	EGYAFEH	250–256	EGYALPH

*This subject sequence made a match with more than one section of the SARS-CoV-2 sequence.

Other SARS-CoV-2 proteins also shared an impressive degree of identity with F-actin protein. An almost similar number of peptide sequences with identity percentages ranging from 33-49% were also detected but are not shown in this table. We also found that some peptide sequences matched with more than one section of SARS-CoV-2; for instance, the actin sequence SIL—ASLSTF shown in this table reacting with the sequence SVLYNSASFSTF in Chain E, SARS-Cov-2 receptor binding protein also cross-reacted with in the SARS-CoV-2 spike protein Chains A, B, C and E, as well as in Chain E of the SARS-CoV-2 spike receptor binding domain, as shown in **Table 4**.

Peptide mapping was done using the NIH/US National Library of Medicine’s BLAST (Basic Local Alignment Search Tool) sequence matching program.

**Table 6:** Potential cross-reactive epitopes between SARS-CoV-2 proteins and TPO antigen.

SARS-CoV-2 antigen	SARS-CoV-2 sequence	Mapped start to end	TPO sequence	ID (%)
Chain A, Helicase	VLTSHT-VM	226–233	VL-SVTLVM*	67
Chain A, Spike protein S1, S2	VLGQSKR-VD	1,020–1,028	VLEESKRLVD*	70
Chain B, Replicase polyprotein 1a	EDKRAKVTSAM-QTM	2–15	ESKRL-VDTAMYATM*	53
Chain A, Spike glycoprotein	NLTTRTQLPPA	48–58	NLKKRGILSPA*	55
Chain A, nsp16	APGTAVLRQWLP	79–90	ASNTALAR-WLP*	58
Chain A, Spike protein S1, S2	VTWFHAIHVS	49–58	VTR-HVIQVS*	60
Chain A, Replicase polyprotein 1ab	SAARLTPCGTG	6–16	SAA—CGTG*	64
Chain A, Replicase polyprotein 1a	LLSVLQQLR	13–21	LLRVHARLR*	56
Chain A, Spike glycoprotein	FL—GRSLEV	1,221–1,228	FLAGDGRASEV	55
Chain A, nsp3	ADIV-EEAKKV	21–30	ADAVYQEARKV*	64
Chain A, Main protease	LNGLWLDDTVY	27–37	LNAHWSADAVY*	55
Chain A, Nucleoprotein	PY-G—AN	77–81	PYEGYDSTAN*	50
Chain A, Spike glycoprotein	WVLLSTFLGRSGGGL	1,219–1,233	WTLL—–R-GGGL	53
Chain A, Spike glycoprotein	VLYNSASFST	34–43	VLSNS—ST	60
Chain A, Uridylate-specific endoribonuclease	SSGVDLGTENL	8–18	SSTLDLASINL	55
Chain A, Uridylate-specific endoribonuclease	TENLYFQSNMS	15–25	TERLFVLSNSS	55
Chain A, 2′-O-methyltransferase	FV-SDADSTL	105–113	FVLSNS-STL*	60
Chain A, Helicase	LSYGIATVREV	147–1,578	LSTAIAS-RSV*	55
Chain A, Spike protein S1, S2	PD-VD–LG	86–91	PDNIDVWLG	56
Chain B, nsp3	RARAGEAANF-CALI	138–151	RARTG–PLFAC-LI*	53
Chain A, Spike glycoprotein	YEQSGRENL	1,237–1,245	YELQGREQL*	67
Chain A, Spike glycoprotein	YKLPDDFTG–CV	90–100	YELGDD—GRTCV*	54
Chain A, 3C-like proteinase	ELLQNGMNGRT	268–278	EL—GDDGRT*	55
S protein in complex bound with 4A8	SPRRARSVASQ	671–681	SPQRA—AAQ*	55

*This subject sequence made a match with more than one section of the SARS-CoV-2 sequence.

SARS-CoV-2 proteins shared an impressive degree of identity with TPO protein. In fact, about twice the number of peptide sequences with identity percentages ranging from 33% to 49% were also detected but are not shown in this table. We also found that some peptide sequences matched with more than one section of SARS-CoV-2; for instance, the TPO sequence VLEESKRLVD shown in this table reacting with the sequence VLGQSKR-VD in Chain A, Spike protein S1, S2 also cross-reacted with other SARS-CoV-2 protein sequences.

Peptide mapping was done using the NIH/US National Library of Medicine’s BLAST (Basic Local Alignment Search Tool) sequence matching program.

### Reaction of Sera Containing No Levels, Low Levels, or High Levels of Mitochondrial Antibodies With Mitochondrial M2 Antigen and SARS-CoV-2 Spike Proteins and Nucleoproteins

We first found that the calibrator and positive controls with known levels of M2 antibody from the Trinity Biotech M2 antibody kit reacted moderately with both SARS-CoV-2 spike proteins and nucleoproteins. In addition, we found that all four sera with elevated M2 antibody also reacted moderately with those same SARS-CoV-2 proteins. The four sera with no detected levels of M2 antibody did not react with those proteins (**Table 7**). We compared the OD values of the reaction of the four positive patients with the ODs of the four negative patients, and the results were statistically significant (**Table 7**). These results further support mimicry between SARS-CoV-2 and M2 proteins resulting in the production of cross-reactive antibodies.

**Table 7:** Reaction of calibrators and controls from mitochondrial M2 antibody kit as well as four sera with negative levels and four sera with positive levels of M2 antibody with M2 antigen-coated wells, SARS-CoV-2 spike protein-coated wells, and SARS-CoV-2 nucleoprotein-coated wells.

OD of calibrators and controlsused in M2 antibody kit		Reaction of sera withM2-antigencoated wells		Reaction of M2 antibody kit controls with SARS-CoV-2 antigen-coated wells			Reaction of 4 different sera with negative M2 antibody with SARS-CoV-2 antigen-coated wells		Reaction of 4 different sera with positive M2 antibody with SARS-CoV-2 antigen-coated wells	
		Negative M2 ab	Positive M2 ab		Spike protein	Nucleoprotein	Spike protein	Nucleoprotein	Spike protein	Nucleoprotein
Blank	0.06	0.213	1.382	Kit Neg Control	0.118	0.214	0.114	0.118	0.491	0.415
		0.241	1.434.		0.137	0.198	0.132	0.126	0.514	0.428
Calibrators	0.981	0.316	1.131	Kit Calibrator	0.489	0.513	0.212	0.183	0.465	0.452
	1.062	0.291	1.244		0.479	0.498	0.189	0.167	0.458	0.463
	1.011	0.178	1.861	Kit Low Positive	0.538	0.413	0.082	0.153	0.618	0.553
Neg Ctrl	0.135	0.163	1.932		0.546	0.422	0.094	0.124	0.643	0.536
Low Pos	1.264	0.218	0.972	Kit High Positive	0.612	0.484	0.178	0.141	0.386	0.378
High Pos	1.975	0.226	0.958		0.594	0.476	0.155	0.123	0.395	0.369

All determinations were performed in duplicate.

Note that while the negative controls from the M2 antibody kit and all four sera with negative M2 antibody did not react with both SARS-CoV-2 spike proteins and nucleoproteins, the kit calibrators, low and high positives plus the four sera with elevated M2 antibody reacted moderately with both SARS-CoV-2 spike proteins and nucleoproteins. When the ODs of the reaction of four sera negative for M2 antibody with SARS-CoV-2 spike proteins and nucleoproteins were compared with the ODs of the reaction of the four sera positive for M2 antibody with the same SARS-CoV-2 proteins, the resulting p values were statistically significant (p <0.0001).

## Discussion

There have been more than 7,000 peer-reviewed studies published on molecular mimicry and autoimmune diseases and over 50 recognized cross-reactive relationships between specific viral pathogens and human tissue proteins (1, 4, 8, 14–20). With the recent global outbreak of SARS-CoV-2, there has been an increased interest in understanding the multitude of diseases that are associated with this new virus and how they may potentially impact the human body. Several articles have remarked on the phenomena of molecular mimicry between SARS-CoV-2 and human proteins, and have postulated a connection between this mimicry and multi-organ disorders beyond the respiratory tract (5, 9, 10, 21–23). The reasoning is that immune response against the viral antigens following infection or vaccination can cross-react with human tissue antigens that share sequence homology with the virus, resulting in autoimmune reactivity, possibly followed by outright autoimmune disease (5, 10, 19, 24, 25). Some support for this proposed mechanism for the induction of autoimmunity was presented by Lyons-Weiler (5) when he compared immunogenic epitopes of SARS-CoV-2 to human proteins and found a high degree of homology with various tissues. These included heart muscle, skeletal muscle, thyroid gland, kidney, brain, pituitary gland, testes, lung, blood, gastrointestinal tract, eye, liver, bone marrow, adipose tissue, skin, and many ubiquitous proteins (5). While our own list of 55 selected human tissue antigens shares some unavoidable overlap with those used in the Lyons-Weiler study (5), we went further and based our selection on key target human tissue proteins that were known to be involved both with extra-pulmonary manifestations of COVID-19 and common autoimmune diseases. For example, the Lyons-Weiler study examined heart muscle, skeletal muscle and thyroid gland, whereas we studied alpha-myosin, actin and TPO. Lyons-Weiler studied brain, liver, GI tract and skin, whereas we examined SARS-CoV-2 cross-reactivity with brain tissue antigens (MBP, NFP, amyloid-beta, alpha-synuclein, synapsin, tTG-6), liver microsomal peptide, M2 protein, PDH peptide, and skin antigens (tTG-2, tTG-3, epithelial cell antigens). We also examined many other tissue antigens, such as barrier proteins, that were not tested in the Lyons-Weiler study. In an effort to provide further proof for this concept, we sought to determine in this study whether human monoclonal antibody that mimics natural antibodies produced by the immune system to fight the SARS-CoV-2 virus will react to various human tissue antigens. This immune reaction may be responsible for the multi-organ system disorder found in patients with severe COVID-19.

In an earlier, limited study that was published in Clinical Immunology (21), we used mouse monoclonal antibody and rabbit monoclonal antibody against SARS-CoV-2 proteins to investigate this possible connection. At that time, the human antibodies we wanted were not commercially available, but the animal antibodies that were available showed cross-reactivity with 11 to 13 human tissue antigens. Our present study used human monoclonal antibodies against SARS-CoV-2 proteins, and we found reactivity with 28 out of 55 tested human antigens. The difference in the number of reactive human antigens stems from the fact that the process of making human monoclonal antibodies is completely different from the classical method for producing mouse or rabbit monoclonal antibodies (26). This cross-reactivity with so many antigens raises important clinical concerns. First, these cross-reactive relationships may play a role in the systemic inflammatory nature of COVID-19. Second, many patients who suffer from viral respiratory distress syndrome continue to suffer from disability and impaired quality of life after recovering from the infection, which may be associated with autoimmunity (27). Third, understanding the relationship of SARS-CoV-2 with autoimmunity can help predict potential adverse reactions from experimental antibody drugs or vaccine development and use. In particular, these antibody drugs have been prominent in recent news cycles as of this writing, even being reportedly used to treat the president. This is why we feel that we should note in particular that two of the anti-SARS-CoV-2 antibodies that we used in this study were in fact human monoclonal antibodies that are identical with the natural antibodies produced by the human body’s immune system. Fourth, cross-reactivity may play a role as a risk factor for the progression of COVID-19 into multi-system disorders. This possibility is most apparent with our findings of cross-reactivity between immune barrier proteins and the viral antigens.

Our study found immune reactivity between SARS-CoV-2 antibodies and barrier target proteins; occludin+zonulin, beta-catenin, and S100B. These proteins are responsible for maintaining the integrity of the barriers. These cross-reactive interactions may lead to permeability of the lung barrier, gut-barrier, and the blood-brain barrier in susceptible individuals (28–31). A recent systematic review and meta-analysis has identified age, smoking, diabetes, cardiovascular disease, and respiratory diseases as significant risk factors associated with increased mortality rate and greater risk for critical illness from COVID-19 (32). Every single one of these identified risk factors is also associated with permeability of the immune barrier systems (33–37). Permeability of the immune barriers may be the essential centerpiece risk factor that is associated with COVID-19 severity, and part of this mechanism may also be associated with the combined impact of cross-reactivity of SARS-CoV-2 with immune barrier proteins. Permeability of these barriers may increase the spread of the virus throughout the body and potentially promote a systemic cytokine storm (38–40). Additionally, permeability of the immune barriers is also an independent mechanism that may promote immune dysregulation and the onset of autoimmune diseases (41). This is of great concern since autoantibodies to phospholipids have been found with COVID-19 and can lead to life-threatening complications of coagulopathy (3).

In addition to the reaction of the SARS-CoV-2 antibodies with tight junction proteins, the human monoclonal antibodies made against spike protein and nucleoprotein reacted with transglutaminase-2 (tTG-2), an enzyme in the intestinal mucosa that plays a role in celiac disease. Moreover, rabbit polyclonal antibody made against SARS-CoV-2 envelope protein reacted strongly with intestinal epithelial cell antigens. This cross-reaction between SARS-CoV-2 and gastrointestinal tissue antigens may be responsible for the gastrointestinal manifestations of COVID-19 (42).

Our study also identified several cross-reactive interactions that may lead to specific autoimmune patterns. For example, we found that SARS-CoV-2 spike protein, nucleoprotein, and membrane protein all cross-reacted with TPO. Furthermore, we found through BLAST sequence matching that many TPO peptide sequences shared homology or similarity with sequences in various SARS-CoV-2 proteins. These findings suggest that antibodies developed against SARS-CoV-2 may promote autoimmune thyroiditis. A recent case study identified sub-acute thyroiditis after SARS-CoV-2 infection (43). It is possible this may have been the first reported case of thyroid cross-reactivity from COVID-19. While data on thyroid pathophysiology is currently not available for COVID-19, patients with SARS have been found to have destruction of thyroid follicular cells, and there are highly similar genomic sequences between SARS-CoV and SARS-CoV-2 (33, 44). Many infections have been associated with the onset of autoimmune thyroid disease from molecular mimicry (4). It is possible that SARS-CoV-2 cross-reactivity with thyroid target proteins could also lead to the onset of autoimmune thyroid disease. Further research will need to be conducted to determine if this relationship exists.

Several SARS-CoV-2 antibody cross-reactions were identified with central nervous system target proteins that included NFP, MBP, GAD-65, beta-amyloid, alpha-synuclein, synapsin and tTG-6. Compared to the other tissue antigens, NFP had the strongest reaction (very strong) with spike protein, and a very strong reaction with membrane protein that was second only to the reaction of membrane protein with M2 antigens. Antibodies against these neural protein targets are detected in patients with neuroautoimmune disorders such as multiple sclerosis, Alzheimer’s disease (AD), and ataxia (45, 46). A high level of neurofilament light chain proteins, which is a marker of neural injury, was detected in COVID-19 patients (47).

At present, we do not know if the reaction of spike protein antibody with five different neuronal antigens and nucleoprotein antibody with three antigens contribute to the neurological complications and neuropsychiatric symptoms that have recently been described in numerous publications (48–50). Coronaviruses have viral characteristics of being extremely neuro-invasive, with the ability to induce direct damage to the central nervous system *via* T cells and complement activation (51). However, antibody cross-reactivity with viral antigens is also an established feature of the onset of neurological autoimmune diseases (52). Further research to evaluate the pathophysiological role of SARS-CoV-2 on the nervous system will need to consider both direct viral-induced pathology and potential antibody immune reactivity through cross-reactivity.

SARS-CoV-2 cross-reactivity was also identified with target proteins to both striated and smooth muscles including actin and alpha-myosin. Actin is a major component of muscle that contributes to the tissue’s contractile property. Alpha-myosin is a heart muscle-specific constrictive protein. We did epitope mapping with BLAST and found many instances of peptide similarity and homology between F-actin and various SARS-CoV-2 proteins at identity percentages of 58% to 63%, with multiple instances and repeats of matches with sequences in different SARS-CoV-2 proteins. At this point, we do not know whether cross-reaction by SARS-CoV-2 spike protein and nucleoprotein with heart and other muscle-related proteins shown in this study is responsible for the cardiovascular manifestations of COVID-19 (53, 54), but this would be a good subject to explore in the future.

Due to the similarity between mitochondrial dysfunction and the induction of multi-organ disorder by SARS-CoC-2, we measured the reactivity of four different antibodies made against SARS-CoV-2 proteins with M2, which is part of the pyruvate dehydrogenase complex. Antibody against this mitochondrial antigen is detected in 90% to 95% of patients with primary biliary cirrhosis (PBC), and occasionally in other liver diseases and scleroderma (55). In fact, mitochondrial M2 was the only antigen out of 28 cross-reactive antigens in our study that had strong to very strong reactions with all four SARS-CoV-2 protein antibodies (**Table 1**). Furthermore, we found that sera negative for M2 antibody had no reactivity with the SARS-CoV-2 proteins, while sera positive for M2 antibody had moderate reactions (**Table 6**). These results further support mimicry between SARS-CoV-2 and M2 proteins resulting in the production of cross-reactive antibodies. Interestingly, we also tested the human monoclonal antibodies made against the viral spike and nucleoprotein with liver microsomal antigens as well as pyruvate dehydrogenase peptide E2 (PDC-E2) subunit; the modification of this subunit by xenobiotics is held to be responsible for the induction of PBC. While we do not know how mitochondrial antibodies contribute to the etiopathology of disease induced by SARS-CoV-2, cross-reaction by SARS-CoV-2 protein antibodies with M2, PDC-E2 and liver microsomal antigens gives further support to a possible role for SARS-CoV-2 in liver autoimmunity. Our own findings strengthen the recent findings by Wang et al (56). that SARS-CoV-2 infection of the liver is an important factor in hepatic impairment in patients with COVID-19. We also did epitope mapping by BLAST for mitochondria M2 against SARS-CoV-2 proteins, and found that mitochondria M2 sequences had even greater similarity and homology with SARS-CoV-2 proteins than actin, especially for spike protein, again with multiple repeats in the same peptide chains as well as sequence matches in different kinds of SARS-CoV-2 proteins.

Recently, Holder and Reddy (57) showed how interaction between SARS-CoV-2 and immune cells alters mitochondrial activities in host cells, providing a receptive intracellular environment for viral replication in infected cells that may contribute to the progression of the disease in COVID-19 patients (57). Additionally, Schreiner et al. showed that in patients with myalgic encephalomyelitis/chronic fatigue syndrome, mitochondria were strongly fragmented by human herpesvirus 6 (HHV-6) and HHV-7; this is believed to be the trigger of the disease (58). Whether such fragmentation of mitochondria that results in the production of mitochondrial antibody occurs with SARS-CoV-2 certainly deserves future investigation.

Lastly, we identified cross-reactivity with autoimmune target proteins involved in mixed connective tissue diseases (MCTD) that included nuclear antigen (NA), extractable nuclear antigen (ENA), histone and collagen. SARS-CoV-2 spike protein antibody reacted with ENA, NA and histone, nucleoprotein antibody reacted with NA, histone and collagen, and membrane protein antibody reacted with histone and collagen.

**Figure 7:**
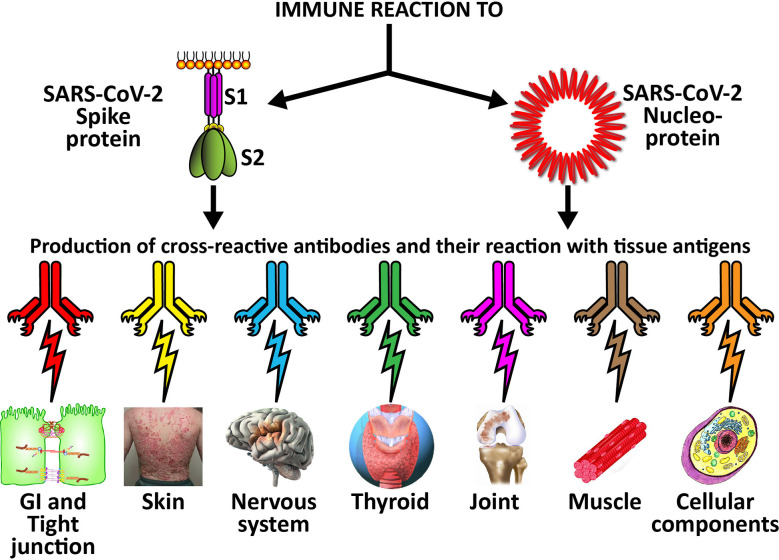
Possible relationship between SARS-CoV-2 proteins and autoimmune target proteins. The cross-reactive patterns between SARS-CoV-2 proteins and autoimmune target proteins may play a role in the systemic inflammatory response from COVID-19, lead to the development of autoimmune diseases post-infection in susceptible subgroups, or potentially play a role in the severity of COVID-19 illness.

The cross-reactive patterns between SARS-CoV-2 proteins and autoimmune target proteins may play a role in the systemic inflammatory response from COVID-19, lead to the development of autoimmune diseases post-infection in susceptible subgroups, or potentially play a role in the severity of COVID-19 illness (see **Figure 7**). When Lyons-Weiler (5) compared human tissues with SARS-CoV-2 for cross-reactivity, he found that most of his identified human target proteins had low overall homology, but high local homology over short segments of their epitopes. His results noted that numerous proteins were expressed in a variety of tissues (5). He also stated that the SARS-CoV-2 spike protein is known to play a role in neuroimmunopathology, but that the SARS-CoV-2 virus has numerous other proteins and polyproteins, any of which could serve as an antigen source during infection leading to autoimmunity (5). Our own BLAST sequence research only focused on mitochondria M2, actin and TPO, but we already found a multiplicity of protein sequences from these three human tissue antigens that found matches in various SARS-CoV-2 proteins, many repeating in different instances and sequences in different subunits of the viral proteins. As Lyons-Weiler said, any of these sequence or epitope matches could potentially lead to autoimmunity by cross-reacting with SARS-CoV-2 antibodies (5). The plethora of these matches between SARS-CoV-2 sequences and human tissues may explain why monoclonal antibodies made against SARS-CoV-2 proteins reacted with so many tissue antigens out of the 55 in our study. It should be noted that our study was limited to the identification of general cross-reactive antibody responses, and our BLAST search was just limited to three human tissues. The results may indicate that the SARS-CoV-2 antibodies reacted against conformational epitopes in the tissue antigens. Our study design did not specifically include analyses that would capture conformational or non-linear epitopes, but any of the tissue sequences that found matches with the viral sequences, especially the highly recurring ones, could possibly be conformational epitopes. Conformational epitopes are not only important in the production of monoclonal neutralizing antibodies, they could also be major targets of autoantibody production in autoimmune diseases (59, 60). Other antigens among our list that had moderate reactions or greater with SARS-CoV-2 may also have sequences here and there in their structure that could potentially be triggers of autoimmunity, and likewise deserve additional attention and study. Further investigation to identify the specific cross-reactive epitopes will require specific peptide fragment inhibition studies as well as computational modeling. More precise identification of conformational autoepitopes is needed to clarify the role of SARS-CoV-2 in autoimmunity.

Sequence homology is also the basis for molecular mimicry, an evolutionary strategy adopted by viruses to exploit the host cellular machinery. In a very recent article (23), Anand et al. reported that SARS-CoV-2 had evolved a unique S1/S2 cleavage site, resulting in striking mimicry of an identical 8-mer FURIN-cleavable peptide on the human epithelial sodium channel α-subunit (ENaC-α). Furin is expressed with ACE2 and ENaC-α across multiple cell types, including the intestine, pancreas and lungs. Further research is needed to determine whether this mimicry is the reason that the SARS-CoV-2 proteins reacted with so many human tissue antigens in our study.

Another concern from molecular mimicry is the potential role it may play in vaccine safety. Several incidences of viral infection and vaccine-induced autoimmunity specific to cross-reactivity have been reported in the literature (8). In 2009, the vaccines developed to treat the H1N1 pandemic lead to narcolepsy specifically due to cross-reactivity. The inactivated split-viron particles (ASO3) shared cross-reactive homology with hypocrites found in the hypothalamus, leading to selective destruction of that substance after vaccination in a subgroup of susceptible individuals (13). Vaccination with ASO3 lead to a three-fold increase in the onset of narcolepsy compared to individuals who were not vaccinated (61).

During the swine flu outbreak in the late 1970s in the United States, the use of influenza vaccination was found to induce a four- to eight-fold increased risk of developing Guillain-Barré syndrome due to cross-reactivity (14). Cross-reactive relationships between viral infections and vaccinations have also been found with hepatitis B and myelin proteins leading to multiple sclerosis, human papillomavirus and nuclear proteins leading to systemic lupus erythematosus (SLE), coxsackievirus and islet cells proteins leading to type 1 diabetes, etc (15–18). Razim et al (62)., in designing a vaccine against Clostridium difficile, concluded that before considering a protein as a vaccine antigen, special care should be taken to analyze and remove the sequences of tissue cross-reactive epitopes in order to avoid possible future side effects.

In a very recent publication in JAMA, Trogen et al. said, “What cannot and must not be allowed is for desperation to result in the suspension of scientific principles and ethical research values (63).” We ourselves would apply these principles and ethical values towards investigating whether SARS-CoV-2 peptides contained in a future vaccine may cross-react with human tissue antigens and possibly result in autoimmunity. But while the possibility of future autoimmune disease is daunting and very real, it must be remembered that without vaccinations the SARS-CoV-2 pandemic will spread unchecked, bringing with it a slew of multiple system disorders including autoimmunities both in the present and the future. We hope that the recently approved human monoclonal antibodies and vaccines can prevent the many extra-pulmonary manifestations and other disorders brought about by COVID-19, and eventually help bring an end to this pandemic.

## Data Availability Statement

The original contributions presented in the study are included in the article/supplementary material. Further inquiries can be directed to the corresponding author.

## Author Contributions

EV conceptualized the study. AV designed the experiments, performed the ELISA assays, and performed the data analysis. EV also performed the ELISA assay that showed cross-reaction between human serum containing M2 antibody and SARS-Cov-2 spike proteins and nucleoproteins. AV and DK wrote the manuscript. DK helped with the data analysis and with the editing of the manuscript. All authors contributed to the article and approved the submitted version.

## Conflict of Interest

AV is the co-owner, CEO and employee of Immunosciences Lab., Inc. EV is the owner and employee of Regenera Medical, a private medical practice.

The remaining authors declare that the research was conducted in the absence of any commercial or financial relationships that could be construed as a potential conflict of interest.
